# Clinical impact of airflow obstruction after allogeneic hematopoietic stem cell transplantation

**DOI:** 10.1007/s12185-024-03831-y

**Published:** 2024-08-27

**Authors:** Sanshiro Nakao, Shokichi Tsukamoto, Yusuke Takeda, Chikako Ohwada, Chihiro Ri, Shintaro Izumi, Yuri Kamata, Shinichiro Matsui, Asuka Shibamiya, Arata Ishii, Koji Takaishi, Kohei Takahashi, Yuki Shiko, Nagisa Oshima-Hasegawa, Tomoya Muto, Naoya Mimura, Koutaro Yokote, Chiaki Nakaseko, Emiko Sakaida

**Affiliations:** 1 Department of Hematology, Chiba University Hospital, 1-8-1 Inohana, Chuo-Ku, Chiba, 260-8677 Japan; 2 Blood and Marrow Transplant Center, Chiba University Hospital, Chiba, Japan; 3 Department of Endocrinology, Hematology and Gerontology, Chiba University Graduate School of Medicine, Chiba, Japan; 4 Department of Hematology, International University of Health and Welfare, Narita, Japan; 5 Biostatistics Section, Clinical Research Centre, Chiba University Hospital, Chiba, Japan; 6 Department of Transfusion Medicine and Cell Therapy, Chiba University Hospital, Chiba, Japan

**Keywords:** Airflow obstruction (AFO), Bronchiolitis obliterans syndrome, Chronic graft-versus-host disease, Allogeneic hematopoietic stem cell transplantation

## Abstract

Criteria for airflow obstruction (AFO) at one year after allogeneic hematopoietic stem cell transplantation (allo-HSCT) in pulmonary function tests (PFTs) are more stringent than the bronchiolitis obliterans syndrome (BOS) criteria of the National Institutes of Health. This single-center, retrospective cohort study evaluated the clinical impact of the AFO criteria at any time after transplantation. In 132 patients who underwent allo-HSCT from 2006 to 2016, the 2-year cumulative incidence of AFO was 35.0%, and the median time to diagnosis of AFO was 101 days after transplantation (range 35–716 days). Overall chronic graft-versus-host disease (cGVHD) incidence was significantly higher in patients with AFO than in those without AFO (80.4% vs. 47.7%, *P* < 0.01); notably, 37.0% of patients with AFO developed cGVHD after AFO diagnosis. AFO patients developed BOS with a 5-year cumulative incidence of 49.1% after AFO onset. The 5-year cumulative incidence of non-relapse mortality in the AFO group was higher than that in the non-AFO group (24.7% vs. 7.1%, *P* < 0.01). These results suggest that closely monitoring PFTs within two years after allo-HSCT, regardless of cGVHD status, is important for early detection of AFO and prevention of progression to BOS. (192words).

## Introduction

Bronchiolitis obliterans syndrome (BOS) is a common late-onset noninfectious pulmonary complication of allogeneic hematopoietic stem cell transplantation (allo-HSCT) that remains associated with high mortality [1–6]. In 1982, Roca et al. reported bronchiolitis obliterans (BO) as a progressive, irreversible obstructive ventilation defect associated with chronic graft-versus-host disease (GVHD) [7]. The 2005 National Institutes of Health (NIH) consensus diagnostic criteria recommend that biopsy-proven BO should be considered a pulmonary lesion of chronic GVHD when the following pulmonary function tests (PFTs) criteria were met: forced expiratory volume in one second per forced vital capacity (FEV_1_/FVC) ratio < 0.7 and predicted forced expiratory volume in one second (pFEV_1_) < 75% [8]. Since lung biopsy is invasive, the 2014 revision of the NIH criteria no longer requires pathologic demonstration to diagnose BOS [9]. The revised criteria emphasize PFTs, with additions such as pFEV_1_ < 75% with ≥ 10% decline over less than two years. However, BOS is still considered irreversible, and the early detection and diagnosis of obstructive airway lesions followed by appropriate intervention are important for improving the prognosis of BOS. Therefore, various evaluation methods have been proposed for the early detection of BOS [10–19].

In 2003, Chien et al. proposed the airflow obstruction (AFO) criteria, which were defined by PFTs one year after transplantation, and stated that pFEV_1_ decline was more than 5% per year and post-transplant FEV_1_/FVC ratio < 0.8. They reported an AFO incidence rate of 26% one year after transplantation and that AFO was associated with increased mortality [20]. As the criteria were more stringent than the NIH BOS criteria, it may be helpful for the early detection of obstructive airway lesions. However, the clinical significance of AFO criteria, if indicated at any time after transplantation, has yet to be appreciated. In this study, we performed a retrospective, single-center study to evaluate the implications of the AFO criteria at any time after transplantation as a predictor of BOS development and for the prognosis by examining the evolution of PFTs in patients undergoing allo-HSCT.

## Materials and methods

### Patient selection

Of 215 patients who underwent allo-HSCT at Chiba University Hospital from April 2006 to December 2016, 132 were included in this analysis after excluding cases with early relapse or death within 90 days after transplantation, cases undergoing prior allo-HSCT within 12 months, or cases with missing data of PFTs. This study was conducted under the principles of the Declaration of Helsinki and was approved by the ethics committee of Chiba University Graduate School of Medicine (accession number: M10310). Informed consent was obtained as an opt-out on the website of Chiba University Hospital.

### Diagnosis of AFO and BOS

The clinical diagnosis of AFO was made by PFTs according to the definition (AFO definition of PFT) as described previously by Chien et al. [20, 21] as follows: (i) more than 5% per year of pFEV_1_ decline from baseline pFEV_1_ and (ii) less than 0.8 of FEV_1_/FVC ratio after transplantation. Routine PFTs were performed before transplantation, at three months after transplantation, one year after transplantation, and others as needed at the attending physician's discretion. The result of pre-transplant PFT was considered the baseline lung function. Chien et al. defined AFO based on the result of PFTs one year after transplantation. In contrast, in this study, AFO was diagnosed any time after transplantation when the patients met the AFO definition of PFT.

With the presence of the characteristic symptoms of chronic GVHD, BOS was diagnosed when all of the following criteria were met: (i) FEV_1_/FVC ratio is less than 0.7, (ii) pFEV_1_ is less than 75% and has decreased by more than 10% in less than two years, (iii) absence of active airway infection, (iv) one of the following supporting features of BOS: (a) evidence of air trapping by expiratory computed tomography (CT) scan or small airway thickening or bronchiectasis by high-resolution chest CT, or (b) evidence of air trapping by PFTs: residual volume > 120% of predicted. If a patient already carries the diagnosis of chronic GVHD by virtue of organ involvement elsewhere, then only criteria (i)–(iii) above are necessary to document chronic GVHD lung involvement [9]. Acute and chronic GVHD was diagnosed and graded according to the NIH criteria [7, 9].

### Definitions

Non-relapse mortality (NRM) was defined as death without relapse after transplantation. The first or second remission of acute myelogenous leukemia, the first remission of acute lymphoblastic leukemia, the first chronic phase of chronic myelogenous leukemia, myelodysplastic syndrome excluding refractory anemia with excess blasts or leukemic transformation, and aplastic anemia were defined as standard-risk diseases, whereas others were defined as high-risk diseases.

### Statistical analysis

Logistic regression analysis was used to evaluate risk factors for developing AFO. Categorical variables were compared among groups using Fisher’s exact test. The result of PFTs (continuous variables) between the two groups was compared with a t test at each time point. A one-way repeated measure ANOVA was used to compare three time points, followed by pairwise comparison with Bonferroni correction as a post hoc test. The probability of overall survival (OS) was estimated by the Kaplan–Meier with semi-landmark analysis and compared among groups with the log-rank test. In patients with AFO, the post-transplant day of AFO development is defined as the landmark day; in patients without AFO, the landmark day is defined as 101 days after transplantation, which is the median day of AFO onset in the AFO group. The cumulative incidences of AFO, relapse, NRM, and BOS estimated were calculated by the Gray method, considering death as a competing risk for AFO, relapse, and BOS; relapse as a competing risk for NRM. The cumulative incidences of relapse, NRM, and BOS were estimated using semi-landmark analysis. Univariate and multivariate analyses for NRM were performed using the Fine and Gray proportional regression model.

All *P* values were two-sided, and *P* < 0.05 was considered statistically significant. Factors with *P* < 0.05 in univariate analysis were included in a multivariate analysis. All statistical analyses were performed with EZR (Saitama Medical Center, Jichi Medical University, Saitama, Japan) [22], which is a graphical user interface for R (The R Foundation for Statistical Computing, Vienna, Austria). More precisely, it is a modified version of R commander designed to add statistical functions frequently used in biostatistics.

## Results

### Patient characteristics

Patient characteristics and the status of the development of AFO are summarized in Table 1. The median age at transplantation was 48 years (range 17–66 years). The graft sources comprised bone marrow in 60.6%, peripheral blood stem cells (PBSC) in 18.2%, and cord blood in 21.2%, in which grafts were received from related donors in 29.5% and unrelated donors in 70.5%. A myeloablative and reduced-intensity conditioning regimen was used in 54.5% and 45.5% of patients. A total body irradiation (TBI)-based conditioning regimen was provided in 68.2% of patients and a busulfan (BU) regimen in 29.5%.

**Table 1:** Patient characteristics

	All patients		AFO		Non-AFO	
	(*n* = 132)		(*n* = 46)		(*n* = 86)	
	*N*	%	*N*	%	*N*	%
Age						
Median	48		52		46	
(Range)	(17–66)		(17–66)		(17–65)	
Sex						
Male	76	57.6	31	67.4	45	52.3
Female	56	42.4	21	45.7	35	40.7
Primary disease						
AML	48	36.4	15	32.6	33	38.4
ALL	25	18.9	8	17.4	17	19.8
CML	6	4.5	2	4.3	4	4.7
MDS	19	14.4	8	17.4	11	12.8
ML	21	15.9	11	23.9	10	11.6
MM	5	3.8	1	2.2	4	4.7
Others	8	6.1	1	2.2	7	8.1
Disease risk						
Standard	67	50.8	16	34.8	51	59.3
High	65	49.2	30	65.2	35	40.7
Graft source						
BM	80	60.6	25	54.3	55	64.0
PBSC	24	18.2	14	30.4	10	11.6
CB	28	21.2	7	15.2	21	24.4
Donor relation						
Related	39	29.5	18	39.1	21	24.4
Unrelated	93	70.5	28	60.9	65	75.6
HLA matching						
Match	77	58.3	32	69.6	45	52.3
Mismatch	55	41.7	14	30.4	41	47.7
Conditioning						
MAC	72	54.5	20	43.5	52	60.5
RIC	60	45.5	26	56.5	34	39.5
TBI	90	68.2	30	65.2	60	69.8
Non TBI	42	31.8	16	34.8	26	30.2
Bu	39	29.5	15	32.6	24	27.9
Non Bu	93	70.5	31	67.4	62	72.1
CMV status						
Recipient- donor-	8	6.1	2	4.3	6	7.0
Others	104	78.8	33	71.7	71	82.6
Unknown	20	15.2	11	23.9	9	10.5
Donor → recipient sex combination						
Female → male	35	26.5	15	32.6	20	23.3
Others	97	73.5	31	67.4	66	76.7
Pre-transplant FEV_1_/FVC ratio						
≧ 0.8	82	62.1	23	50.0	59	68.6
< 0.8	40	30.3	23	50.0	17	19.8
Smoking history						
Presence	69	52.3	31	67.4	38	44.2
Absence	63	47.7	15	32.6	48	55.8
Period of transplant						
2006–2011	55	41.7	20	43.5	35	40.7
2012–2016	77	58.3	26	56.5	51	59.3

*AML* acute myelogenous leukemia, *ALL* acute lymphoblastic leukemia, *CML* chronic myelogenous leukemia, *MDS* myelodysplastic syndrome, *ML* malignant lymphoma, *MM* multiple myeloma, *BM* bone marrow, *PBSC* peripheral blood stem cell, *CB* cord blood, *MAC* myeloablative conditioning, *RIC* reduced-intensity conditioning, *TBI* total body irradiation, *Bu* busulfan, *CMV* cytomegalovirus, *FEV*_*1*_*/FVC* forced expiratory volume in 1 s per forced vital capacity

### Incidence of AFO after allo-HSCT

**Fig. 1:**
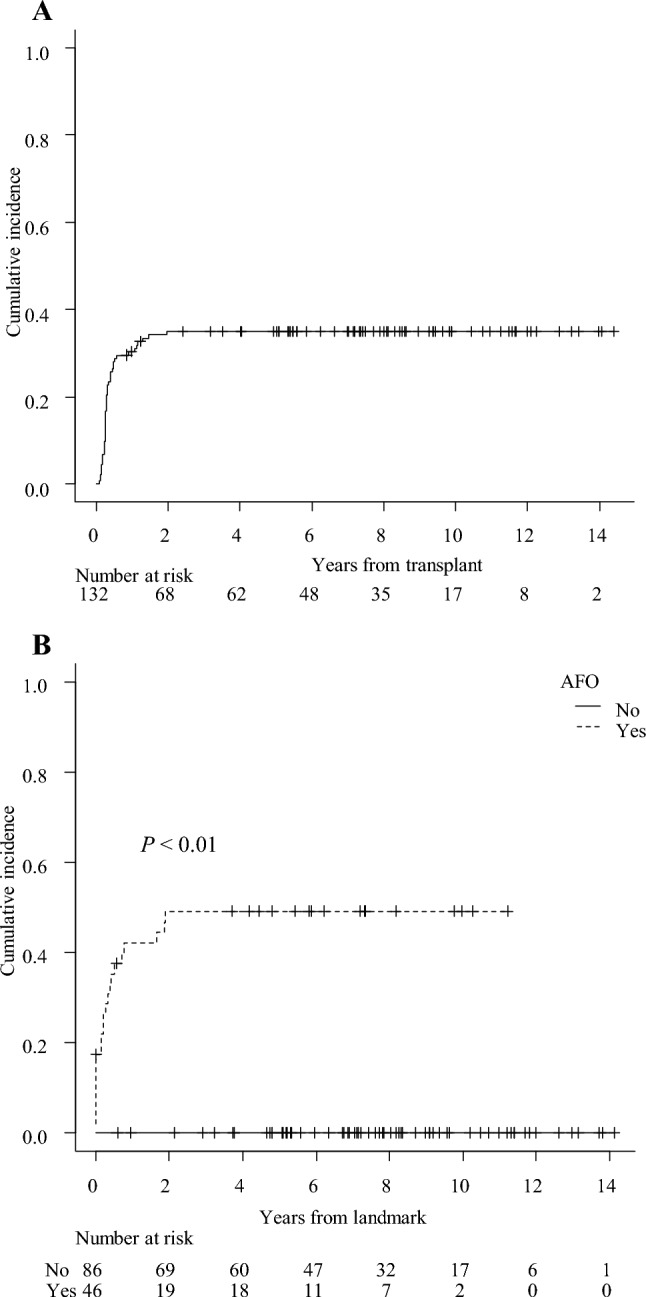
Cumulative incidence of AFO and BOS. **A** A 2-year cumulative incidence of AFO in all the patients was 35.0%, and the median AFO onset was 101 days (range 35–716 days) after transplantation. **B** A cumulative incidence of BOS from landmark days in the AFO and the non-AFO groups. In patients with AFO, the post-transplant day of AFO development is defined as the landmark day; in patients without AFO, the landmark day is defined as 101 days after transplantation, the median day of AFO onset in patients with AFO

**Fig. 2:**
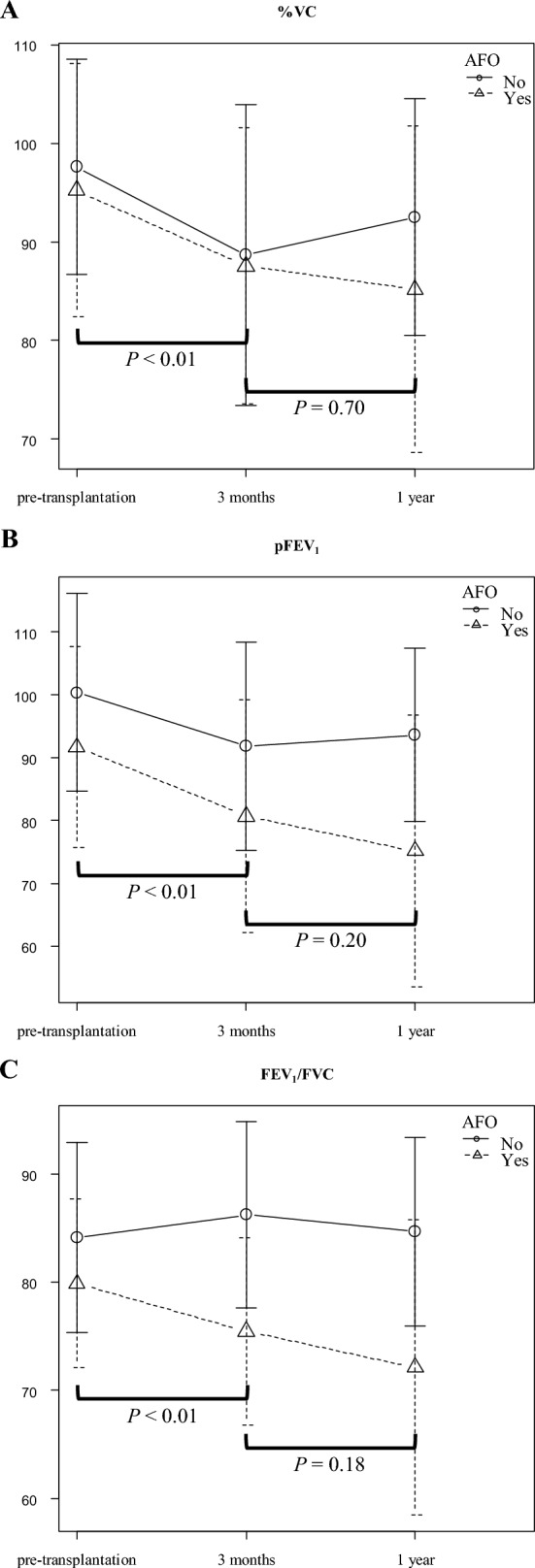
Changes in the results of pulmonary function tests (PFTs) in the AFO and the non-AFO groups. **A** Vital capacity as a percent of predicted (%VC), **B** predicted forced expiratory volume in one second (pFEV_1_), **C** forced expiratory volume in 1 s per forced vital capacity (FEV_1_/FFC) ratio at the pre-transplant, three months after transplantation, and one year after transplantation in the AFO and the non-AFO groups. Results are shown as means ± SD. **A** In the AFO group, the mean %VC at the pre-transplant, the three months after transplantation, and the one year after transplantation was 95.7 ± 13.4%, 86.3 ± 16.6%, and 85.2 ± 16.6%, respectively. **B** In the AFO group, mean pFEV_1_ at the pre-transplant, the three months after transplantation, and the one year after transplantation was 91.9 ± 16.4%, 79.6 ± 19.1%, and 75.1 ± 21.6%, respectively. **C** In the AFO group, the mean FEV_1_/FVC ratio at the pre-transplant, the three months after transplantation, and the one year after transplantation was 79.5 ± 7.8%, 75.2 ± 8.3%, and 72.1 ± 13.7%, respectively. The data were shown in only analyzed patients whose information at all 3 points was available (*n* = 98). The number of patients at the pre-transplant, the three months after transplantation, and the one year after transplantation was 132, 132, and 98, respectively, and analyses using a linear mixed-effects model were performed to exclude the impact of patients with missing data and did not change the results

Forty-six patients met the diagnostic criteria for AFO with a 2-year cumulative incidence of 35.0% after transplantation (Fig. 1A). The median time to AFO diagnosis after transplantation was 101 days (range 35–716 days). PFTs were analyzed at the following three-time points: pre-transplant (median, 26 days before transplantation (day 26); range day 160–8), three months after transplantation (median, 100 days after transplantation (day 100); range day 55–237), and one year after transplantation (median, 361.5 days after transplantation; range day 231–467). In the AFO group, %VC, pFEV_1_, and FEV_1_/FVC significantly decreased at three months after transplantation compared to the pre-transplant (*P* < 0.01). There was a trend decrease one year after transplantation (Fig. 2A, B, C). The pre-transplant pFEV_1_ was significantly lower in the AFO group (mean ± SD, 91.9% ± 16.4%) than that in the non-AFO group (mean ± SD, 99.8% ± 15.5%; *P* < 0.01) (Fig. 2B). Moreover, the pre-transplant FEV_1_/FVC was also significantly lower in the AFO group (mean ± SD, 79.5% ± 7.8%) than that in the non-AFO group (mean ± SD, 84.8% ± 8.0%; *P* < 0.01) (Fig. 2C).

### Risk factors for developing AFO

This study explored the risk factors for developing AFO (Table 2). Age ≥ 50 years at transplantation (odds ratio [OR], 2.19; 95% confidence interval [CI], 1.06–4.55; *P* = 0.03), high-risk disease (OR 2.48; 95% CI 1.18–5.21; *P* = 0.02), PBSC as stem cell source (OR 3.32; 95% CI 1.34–8.27, *P* < 0.01), pre-transplant FEV_1_/FVC ratio < 0.8 (OR 4.06; 95% CI 1.85–8.90; *P* < 0.01) and smoking history before transplantation (OR 2.61; 95% CI 1.23–5.52; *P* = 0.01) were the risk factors for developing AFO in the univariate analysis. In a multivariate analysis of each risk factor extracted in univariate analysis, PBSC as stem cell source (OR 3.42; 95% CI 1.27–9.25; *P* = 0.02) and pre-transplant FEV_1_/FVC ratio < 0.8 (OR 3.26; 95% CI 1.33–7.98; *P* = 0.01) were significant risk factors for developing AFO.

**Table 2:** Univariate and multivariate analysis of risk factors for developing AFO

Factor		Univariate Analysis		Multivariate Analysis	
		*P* value	OR (95% CI)	*P* value	OR (95% CI)
Age	≧ 50 (vs. < 50)	0.03	2.19 (1.06–4.55)	0.40	1.45 (0.61–3.45)
Sex	Male (vs. female)	0.10	1.88 (0.89–3.98)		
Disease risk	High (vs. standard)	0.02	2.48 (1.18–5.21)	0.14	1.89 (0.82–4.36)
Graft source	PBSC (vs. CB, BM)	< 0.01	3.32 (1.34–8.27)	0.02	3.42 (1.27–9.25)
Donor relation	Related (vs. unrelated)	0.08	1.99 (0.92–4.30)		
HLA matching	Match (vs. mismatch)	0.06	2.08 (0.977–4.44)		
Conditioning	RIC (vs. MAC)	0.06	1.99 (0.96–4.11)		
	TBI (vs. non-TBI)	0.59	0.81 (0.38–1.74)		
	Bu (vs. non-BU)	0.57	1.25 (0.58–2.72)		
Pre-transplant FEV_1_/FVC ratio	< 0.8 (vs. ≧ 0.8)	< 0.01	4.06 (1.85–8.90)	0.01	3.26 (1.33–7.98)
CMV status	Recipient- donor- (vs. others, unknown)	0.55	0.61 (0.12–3.13)		
Donor → recipient sex combination	Female → male (vs. Others)	0.25	1.60 (0.72–3.53)		
Smoking history	Presence (vs. absence)	0.01	2.61 (1.23–5.52)	0.25	1.64 (0.71–3.81)

*BM* bone marrow, *PBSC* peripheral blood stem cell, *CB* cord blood, *MAC* myeloablative conditioning, *RIC* reduced-intensity conditioning, *TBI* total body irradiation, *Bu* busulfan, *CMV* cytomegalovirus, *FEV*_*1*_*/FVC* forced expiratory volume in 1 s per forced vital capacity

### Association of acute and chronic GVHD with the AFO development

**Fig. 3:**
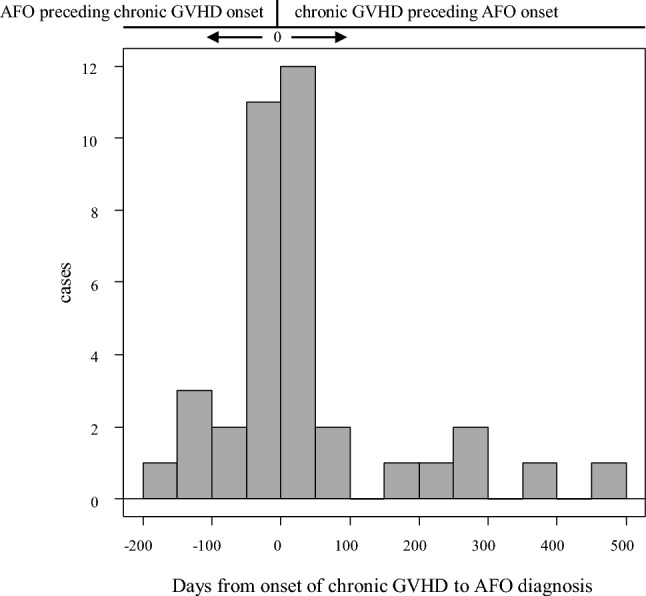
Distribution of time to onset of chronic GVHD in AFO patients. Twenty of 46 patients in the AFO group (43.5%) developed chronic GVHD preceding AFO diagnosis, whereas 17 patients in the AFO group (37.0%) had AFO diagnosis preceding chronic GVHD and the median time to develop chronic GVHD from AFO diagnosis was 41 days (range 1–181 days) in 17 patients

The incidence and severity of acute GVHD were not associated with AFO development (Table 3). Chronic GVHD was observed in 37 of 46 patients with AFO. Therefore, the incidence of chronic GVHD was significantly higher in the AFO group than in the non-AFO group (80.4% vs. 47.7%, *P* < 0.01). In the AFO group, 20 of 46 patients (43.5%) had active chronic GVHD at the time of AFO diagnosis. In contrast, 17 (37.0%) developed chronic GVHD after AFO diagnosis, and the median time to chronic GVHD development after AFO diagnosis was 41 days (range 1–181 days; Fig. 3). The incidence of moderate or severe chronic GVHD in the AFO group was significantly higher than in the non-AFO group (76.1% vs. 27.9%, *P* < 0.01).

**Table 3:** Incidence and severity of acute and chronic GVHD with the development of AFO

	All patients		AFO		Non-AFO		*P* value
	(*n* = 132)		(*n* = 46)		(*n* = 86)		
	*N*	%	*N*	%	*N*	%	
Acute GVHD							
Any grade	90	68.2	32	69.6	58	67.4	0.85
Grade II–IV	64	48.5	23	50.0	41	47.7	0.86
Grade III–IV	10	7.6	3	6.5	7	8.1	1.00
Chronic GVHD							
Any Grade	78	59.1	37	80.4	41	47.7	< 0.01
Moderate–severe	59	44.7	35	76.1	24	27.9	< 0.01

*GVHD* graft-versus-host disease

### Progression to BOS after the onset of AFO

Of the 46 patients diagnosed with AFO, 22 developed BOS with a 5-year cumulative incidence of 49.1% after AFO onset (Fig. 1B), in which eight (17.3% of AFO patients) already met the diagnostic criteria for BOS at the time of AFO diagnosis. None of the non-AFO patients developed BOS. The median time to BOS diagnosis was 229 days after transplantation (range 62–1230 days) and 77 days after AFO diagnosis (range 0–693 days). When we calculated the predictive characteristics of the AFO criteria for the development of BOS, we observed a sensitivity of 100%, a specificity of 78.2%, a positive predictive value of 47.8%, and a negative predictive value of 100%.

### Survival and NRM associated with the development of AFO

**Fig. 4:**
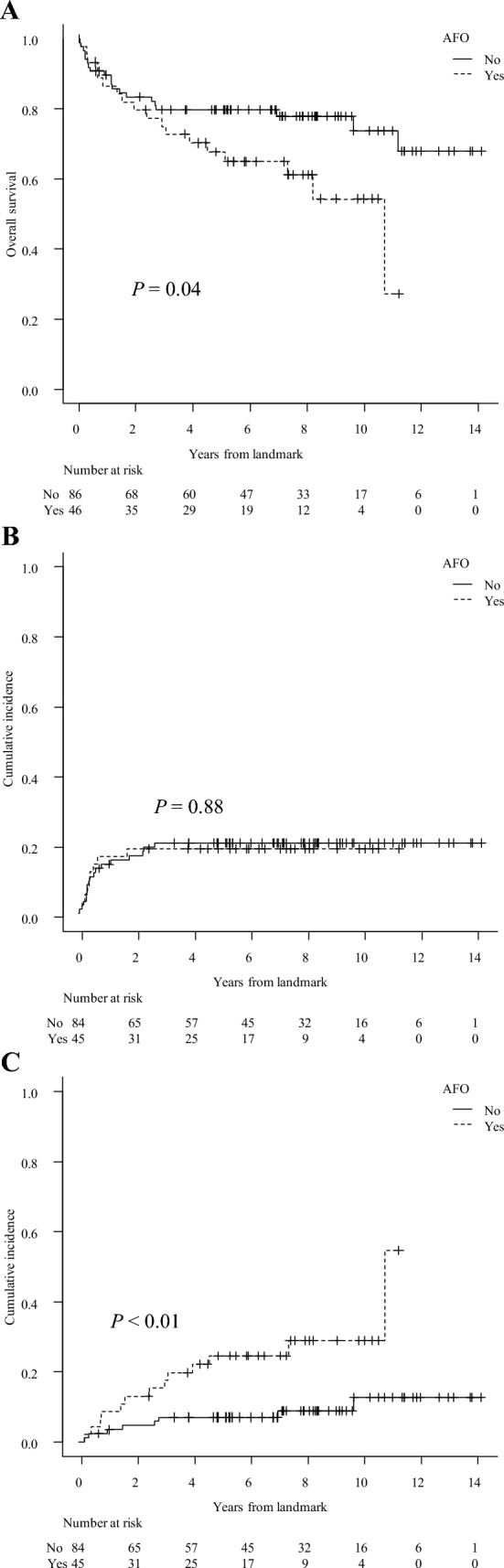
Overall survival (OS), relapse, and non-relapse mortality (NRM) in the AFO and the non-AFO groups. **A** OS from landmark day. In patients with AFO, the post-transplant day of AFO development is defined as the landmark day; in patients without AFO, the landmark day is defined as 101 days after transplantation, the median day of AFO onset in the AFO group. **B** Cumulative incidence of relapse from landmark day. (C) Cumulative incidence of NRM from landmark day

The 5-year OS in the AFO group was significantly inferior to that in the non-AFO group in semi-landmark analysis (67.6% vs. 79.6%, *P* = 0.04) (Fig. 4A). The cumulative incidence of relapse at five years in the AFO group and the non-AFO group was comparable (19.6% vs. 21.1%, *P* = 0.88) (Fig. 4B); the 5-year cumulative incidence of NRM in the AFO group was higher than that in the non-AFO group (24.7% vs. 7.1%, *P* < 0.01) (Fig. 4C). In the AFO group, 18 patients died of respiratory-related diseases (*n* = 6, 33.3%), the primary disease after relapse (*n* = 5, 27.8%), secondary cancers (*n* = 3, 16.7%), and other causes (*n* = 4, 22.2%), whereas in the non-AFO group, 18 patients died of the primary disease after relapse (*n* = 11, 61.1%), secondary cancers (*n* = 3, 16.7%), respiratory-related diseases (*n* = 3, 16.7%), and other causes (*n* = 1, 5.6%).

We then analyzed an NRM risk after transplantation (Table 4). In univariate analysis, AFO development as a time-dependent variable (hazard ratio [HR] 3.95; 95% CI 1.63–9.58; *P* < 0.01), age ≥ 50 years at transplantation (HR 4.61; 95% CI 1.66–12.79; *P* < 0.01), and development of chronic GVHD as a time-dependent variable (HR 3.04; 95% CI 1.19–7.78; *P* = 0.02) were found to have a risk for NRM. In a multivariate analysis, patients who developed AFO after transplantation (HR 2.68; 95% CI 1.04–6.93; *P* = 0.04) or patients aged ≥ 50 years at transplantation (HR 3.40; 95% CI 1.09–10.58; *P* = 0.03) were found to have a significant risk for NRM, whereas development of chronic GVHD as a time-dependent variable was not a risk for NRM. Therefore, although there was a strong association between the development of AFO and chronic GVHD (Table 3), AFO development as a time-dependent variable was an independent risk for NRM, suggesting the importance of close monitoring the PFTs after allo-HSCT for early detection of AFO.

**Table 4:** Univariate and multivariate analysis of NRM

Factor		Univariate analysis		Multivariate analysis	
		*P* value	HR (95% CI)	*P* value	HR (95% CI)
AFO	(vs. non-AFO, time-dependent variables)	< 0.01	3.95 (1.63–9.58)	0.04	2.68 (1.04–6.93)
Age	≧ 50 (vs. < 50)	< 0.01	4.61 (1.66–12.79)	0.03	3.40 (1.09–10.58)
Graft source	PBSC (vs. CB, BM)	0.45	1.47 (0.54–4.04)		
Donor relation	Unrelated (vs. related)	0.59	0.78 (0.31–1.94)		
HLA matching	Match (vs. mismatch)	0.06	2.60 (0.97–6.97)		
Conditioning	RIC (vs. MAC)	0.18	1.78 (0.77–4.13)		
Pre-transplant FEV_1_/FVC ratio	< 0.8 (vs. ≧ 0.8)	0.16	1.84 (0.78–4.32)		
Donor → recipient sex combination	Female → male (vs. others)	0.12	1.98 (0.83–4.75)		
Period of transplant	2006–2011 (vs. 2012–2016)	0.15	1.94 (0.79–4.72)		
acute GVHD	Any Grade (vs. none)	0.11	2.73 (0.80–9.28)		
	Grade II–IV (vs. none–I)	0.58	1.27 (0.54–2.96)		
	Grade III–IV (vs. none–II)	0.23	2.07 (0.63–6.77)		
chronic GVHD	Any grade (vs. none)	0.12	2.21 (0.80–6.08)		
	Moderate–severe (vs. none–mild)	0.04	2.68 (1.06–6.76)		
	Severe (vs. none–moderate)	< 0.01	4.13 (1.78–9.57)		
chronic GVHD	Any grade (vs none, time-dependent variables)	0.02	3.04 (1.19–7.78)	0.24	1.77 (0.68–4.63)

BM, bone marrow; PBSC, peripheral blood stem cell; CB, cord blood, MAC, myeloablative conditioning; RIC, reduced-intensity conditioning, *FEV*_*1*_*/FVC* forced expiratory volume in 1 s per forced vital capacity, *GVHD* graft-versus-host disease

### Impact of therapeutic interventions for AFO on the incidence of BOS

We examined the impact of therapeutic interventions for AFO on the incidence of BOS. In our study, AFO patients received systemic or inhaled corticosteroids, with macrolides or montelukast as therapeutic interventions for AFO. Between 2006 and 2011, one in 12 AFO patients (8.3%) received treatment within three months of AFO diagnosis, whereas between 2012 and 2016, nine in 20 AFO patients (45.0%) received treatment. The cumulative incidence of BOS in the AFO group was 67.2% in 2006–2011 and 35.5% in 2012–2016, suggesting early intervention, including inhaled corticosteroids for AFO might prevent the development of BOS.

## Discussion

Chien et al. proposed the AFO criteria based on the result of PFTs one year after transplantation and reported an AFO incidence rate of 26% [20, 21]. AFO was associated with increased mortality after transplantation; however, since the AFO criteria were more stringent than the NIH BOS criteria, we conducted this study to evaluate its potential usefulness in the early detection of BOS if indicated at any time after transplantation. By modifying the original AFO criteria into the AFO criteria at any time after transplantation in this study, we found a 2-year cumulative incidence of AFO was 35.0%, the median time to diagnosis of AFO was 101 days after transplantation (range 35–716 days), and all AFO patients developed AFO within two years after transplantation. Among 46 patients with AFO, eight already met the diagnostic criteria for BOS at AFO diagnosis, and the median time from AFO diagnosis to BOS onset was 77 days (range 0–693 days). Recently, the NIH consensus project has recommended that all the allo-HSCT recipients (even asymptomatic) undergo regular PFT before transplantation and every three months after that for at least one year [23]. However, we found that the development of AFO occurs until two years after transplantation, suggesting that undergoing PFTs every three months within two years after transplantation was important for early detection of AFO.

Regarding risk factors for developing AFO, PBSC as a stem cell source and pre-transplant FEV_1_/FVC ratio < 0.8 were significant risk factors for developing AFO. Therefore, patients who have these risk factors before transplantation require careful monitoring of PFTs after transplantation. Furthermore, the incidence of chronic GVHD complications in the AFO group was significantly higher than in the non-AFO group in this study (Table 3). Similarly, Chien et al. reported that age at the time of transplant, pre-transplant FEV_1_/FVC ratio, and the incidence of chronic GVHD each conferred a significant risk for the development of AFO [20]. In contrast to a previous study, AFO was diagnosed any time after transplantation when the patients met the AFO definition of PFT in this study. We found that 17 patients in the AFO group (37.0%) developed chronic GVHD after AFO diagnosis. The median time to develop chronic GVHD from AFO diagnosis was 41 days (range 1–181 days) in 17 patients. Therefore, we suggest the importance of close monitoring of the PFTs after allo-HSCT, regardless of the status of chronic GVHD, for early detection of AFO. The 2014 revision of the NIH criteria for BOS can detect obstructive airway lesions by PFTs earlier than biopsy-proven BO in the 2005 NIH consensus diagnostic criteria. However, BOS is still considered irreversible. Several attempts to predict the development of BOS before the diagnosis of BOS have been proposed [10–19]. Jamani et al. recently reported that forced expiratory flow between 25 and 75% of maximum (FEF_25–75_) at 80 days after transplantation might be more critical than FEV_1_ in predicting the development of BOS [11]. Similarly, Turner et al. reported the usefulness of home spirometry tele-monitoring for the early detection of BOS [13]. Moreover, BOS stage 0p (BOS 0p) was reported to be a parameter detected on PFT after lung transplantation to identify BOS in patients at risk. It was also valuable to identify an at-risk population for the development of BOS after allo-HSCT [12]. The definition of BOS 0p is a decline in FEV_1_ of 10% to 19% of predicted normal or a > 25% decline in FEF_25–75_ at any point post-HSCT. A previous study reported an estimated 2-year cumulative incidence of BOS 0p was 16%, and the median time to detection of BOS 0p was 273 days (range 32–1910 days) after allo-HSCT [12]. The sensitivity for the detection of BOS by the BOS 0p criteria was 85%, with a positive predictive value of 29%. In our study, the sensitivity for the detection of BOS by the AFO criteria was 100%, with a positive predictive value of 48%, indicating the usefulness of the AFO criteria for predicting the development of BOS.

Treatment for BOS has not been established, and historically, protracted systemic corticosteroids or other immunosuppressive agents, such as calcineurin inhibitors, have been used for BOS. However, long-term use of these drugs can lead to infectious complications and other adverse events [24]. The efficacy of inhaled steroids and other treatments for BO has been reported since around 2007 [25, 26], and Williams et al. reported that treatment with inhaled fluticasone, azithromycin, and montelukast and a brief steroid pulse stabilized the majority of new-onset BOS patients [27]. This approach is widely used for BOS treatment due to its tolerability [4, 5, 28]. However, the effectiveness of these treatments in patients with AFO before the development of BOS remains unclear. Our study suggested that early intervention, including inhaled corticosteroids for AFO, could prevent the development of BOS. However, outcomes in patients undergoing allo-HSCT have improved with improved prophylaxis of GVHD or application of several antifungal or antiviral agents and supportive care. Therefore, prospective studies are needed to evaluate the efficacy and safety of the early intervention, including inhaled corticosteroids, for AFO.

The major limitation of this study was that it was a single-center, retrospective study with a small sample size. However, it also had some strengths, including the availability of detailed information with long-term follow-up after transplantation. Another limitation is that the treatment method for AFO within three months of AFO diagnosis was not established in our institution, and the treatments were performed on each patient by physicians’ decisions. Further prospective investigation is necessary to confirm the impact of treatment on patients with AFO.

In conclusion, AFO development after allo-HSCT is associated with NRM and poor prognosis. AFO occurs within two years after allo-HSCT in patients with or without chronic GVHD. Therefore, close monitoring of the PFTs, especially within two years after transplantation, is important for early detection of AFO and prevention of progression to BOS. Prospective studies are needed to evaluate the efficacy and safety of early interventions, including inhaled corticosteroids, for AFO.

## Data Availability

Due to the nature of this research, participants of this study did not agree for their data to be shared publicly, so supporting data is not available.

## References

[1] Bergeron A, Chevret S, Peffault De Latour R, Chagnon K, De Margerie-Mellon C, Rivière F, et al. Noninfectious lung complications after allogeneic haematopoietic stem cell transplantation. Eur Respir J. 2018;51(5):1702617. 10.1183/13993003.02617-2017. 29650555 10.1183/13993003.02617-2017

[2] Dudek AZ, Mahaseth H, DeFor TE, Weisdorf DJ. Bronchiolitis obliterans in chronic graft-versus-host disease: analysis of risk factors and treatment outcomes. Biol Blood Marrow Transplant. 2003;9(10):657–66. 10.1016/s1083-8791(03)00242-8. 14569562 10.1016/s1083-8791(03)00242-8

[3] Au BK, Au MA, Chien JW. Bronchiolitis obliterans syndrome epidemiology after allogeneic hematopoietic cell transplantation. Biol Blood Marrow Transplant. 2011;17(7):1072–8. 10.1016/j.bbmt.2010.11.018. 21126596 10.1016/j.bbmt.2010.11.018 PMC3061253

[4] Williams KM. Noninfectious complications of hematopoietic cell transplantation. Hematology. 2021;2021(1):578–86. 10.1182/hematology.2021000293. 34889438 10.1182/hematology.2021000293 PMC8791176

[5] Astashchanka A, Ryan J, Lin E, Nokes B, Jamieson C, Kligerman S, et al. Pulmonary complications in hematopoietic stem cell transplant recipients—a clinician primer. J Clin Med. 2021;10(15):3227. 10.3390/jcm10153227. 34362012 10.3390/jcm10153227 PMC8348211

[6] Shiari A, Nassar M, Soubani AO. Major pulmonary complications following Hematopoietic stem cell transplantation: what the pulmonologist needs to know. Respir Med. 2021;185: 106493. 10.1016/j.rmed.2021.106493. 34107323 10.1016/j.rmed.2021.106493

[7] Filipovich AH, Weisdorf D, Pavletic S, Socie G, Wingard JR, Lee SJ, et al. National Institutes of health consensus development project on criteria for clinical trials in chronic graft-versus-host disease: I. Diagnosis and staging working group report. Biol Blood Marrow Transplant. 2005;11(12):945–56. 10.1016/j.bbmt.2005.09.004. 16338616 10.1016/j.bbmt.2005.09.004

[8] Roca J, Grañeña A, Rodriguez-Roisin R, Alvarez P, Agusti-Vidal A, Rozman C. Fatal airway disease in an adult with chronic graft-versus-host disease. Thorax. 1982;37(1):77–8. 10.1136/thx.37.1.77. 7071798 10.1136/thx.37.1.77 PMC459252

[9] Jagasia MH, Greinix HT, Arora M, Williams KM, Wolff D, Cowen EW, et al. National Institutes of health consensus development project on criteria for clinical trials in chronic graft-versus-host disease: I. The 2014 diagnosis and staging working group report. Biol Blood Marrow Transplant. 2015;21(3):389-401.e1. 10.1016/j.bbmt.2014.12.001. 25529383 10.1016/j.bbmt.2014.12.001 PMC4329079

[10] Bergeron A, Godet C, Chevret S, Lorillon G, Peffault de Latour R, de Revel T, et al. Bronchiolitis obliterans syndrome after allogeneic hematopoietic SCT: phenotypes and prognosis. Bone Marrow Transplant. 2013;48(6):819–24. 10.1038/bmt.2012.241. 23208317 10.1038/bmt.2012.241 PMC7091913

[11] Jamani K, He Q, Liu Y, Davis C, Hubbard J, Schoch G, et al. Early post-transplantation spirometry is associated with the development of bronchiolitis obliterans syndrome after allogeneic hematopoietic cell transplantation. Biol Blood Marrow Transplant. 2020;26(5):943–8. 10.1016/j.bbmt.2019.12.002. 31821885 10.1016/j.bbmt.2019.12.002 PMC7255698

[12] Abedin S, Yanik GA, Braun T, Pawarode A, Magenau J, Goldstein SC, et al. Predictive value of bronchiolitis obliterans syndrome stage 0p in chronic graft-versus-host disease of the lung. Biol Blood Marrow Transplant. 2015;21(6):1127–31. 10.1016/j.bbmt.2015.02.006. 25687798 10.1016/j.bbmt.2015.02.006 PMC4970454

[13] Turner J, He Q, Baker K, Chung L, Lazarevic-Fogelquist A, Bethune D, et al. Home spirometry telemonitoring for early detection of bronchiolitis obliterans syndrome in patients with chronic graft-versus-host disease. Transplant Cell Ther. 2021;27(7):616.e1-e66. 10.1016/j.jtct.2021.03.024. 10.1016/j.jtct.2021.03.024 PMC8423348 33781975

[14] Cheng GS, Selwa KE, Hatt C, Ram S, Fortuna AB, Guerriero M, et al. Multicenter evaluation of parametric response mapping as an indicator of bronchiolitis obliterans syndrome after hematopoietic stem cell transplantation. Am J Transplant. 2020;20(8):2198–205. 10.1111/ajt.15814. 32034974 10.1111/ajt.15814 PMC7395854

[15] Sharifi H, Lai YK, Guo H, Hoppenfeld M, Guenther ZD, Johnston L, et al. Machine learning algorithms to differentiate among pulmonary complications after hematopoietic cell transplant. Chest. 2020;158(3):1090–103. 10.1016/j.chest.2020.02.076. 32343962 10.1016/j.chest.2020.02.076 PMC8097633

[16] Walkup LL, Myers K, El-Bietar J, Nelson A, Willmering MM, Grimley M, et al. Xenon-129 MRI detects ventilation deficits in paediatric stem cell transplant patients unable to perform spirometry. Eur Respir J. 2019;53(5):1801779. 10.1183/13993003.01779-2018. 30846475 10.1183/13993003.01779-2018 PMC6945824

[17] Liu X, Yue Z, Yu J, Daguindau E, Kushekhar K, Zhang Q, et al. Proteomic characterization reveals that MMP-3 correlates with bronchiolitis obliterans syndrome following allogeneic hematopoietic cell and lung transplantation. Am J Transplant. 2016;16(8):2342–51. 10.1111/ajt.13750. 26887344 10.1111/ajt.13750 PMC4956556

[18] Ditschkowski M, Elmaagacli AH, Koldehoff M, Gromke T, Trenschel R, Beelen DW. Bronchiolitis obliterans after allogeneic hematopoietic SCT: further insight–new perspectives? Bone Marrow Transplant. 2013;48(9):1224–9. 10.1038/bmt.2013.17. 23435515 10.1038/bmt.2013.17

[19] Kuzmina Z, Krenn K, Petkov V, Körmöczi U, Weigl R, Rottal A, et al. CD19(+)CD21(low) B cells and patients at risk for NIH-defined chronic graft-versus-host disease with bronchiolitis obliterans syndrome. Blood. 2013;121(10):1886–95. 10.1182/blood-2012-06-435008. 23303823 10.1182/blood-2012-06-435008

[20] Chien JW, Martin PJ, Gooley TA, Flowers ME, Heckbert SR, Nichols WG, et al. Airflow obstruction after myeloablative allogeneic hematopoietic stem cell transplantation. Am J Respir Crit Care Med. 2003;168(2):208–14. 10.1164/rccm.200212-1468oc. 12649126 10.1164/rccm.200212-1468OC

[21] Chien JW, Martin PJ, Flowers ME, Nichols WG, Clark JG. Implications of early airflow decline after myeloablative allogeneic stem cell transplantation. Bone Marrow Transplant. 2004;33(7):759–64. 10.1038/sj.bmt.1704422. 14968136 10.1038/sj.bmt.1704422

[22] Kanda Y. Investigation of the freely available easy-to-use software ‘EZR’ for medical statistics. Bone Marrow Transplant. 2013. 10.1038/bmt.2012.244. 23208313 10.1038/bmt.2012.244 PMC3590441

[23] Kitko CL, Pidala J, Schoemans HM, Lawitschka A, Flowers ME, Cowen EW, et al. National Institutes of health consensus development project on criteria for clinical trials in chronic graft-versus-host disease: IIa. The 2020 clinical implementation and early diagnosis working group report. Transplant Cell Ther. 2021;27(7):545–57. 10.1016/j.jtct.2021.03.033. 33839317 10.1016/j.jtct.2021.03.033 PMC8803210

[24] Williams KM. How I treat bronchiolitis obliterans syndrome after hematopoietic stem cell transplantation. Blood. 2017;129(4):448–55. 10.1182/blood-2016-08-693507. 27856461 10.1182/blood-2016-08-693507 PMC5270387

[25] Bergeron A, Belle A, Chevret S, Ribaud P, Devergie A, Esperou H, et al. Combined inhaled steroids and bronchodilatators in obstructive airway disease after allogeneic stem cell transplantation. Bone Marrow Transplant. 2007;39(9):547–53. 10.1038/sj.bmt.1705637. 17351647 10.1038/sj.bmt.1705637

[26] Bashoura L, Gupta S, Jain A, Couriel DR, Komanduri KV, Eapen GA, et al. Inhaled corticosteroids stabilize constrictive bronchiolitis after hematopoietic stem cell transplantation. Bone Marrow Transplant. 2008;41(1):63–7. 10.1038/sj.bmt.1705877. 17934530 10.1038/sj.bmt.1705877

[27] Williams KM, Cheng G-S, Pusic I, Jagasia M, Burns L, Ho VT, et al. Fluticasone, azithromycin, and montelukast treatment for new-onset bronchiolitis obliterans syndrome after hematopoietic cell transplantation. Biol Blood Marrow Transplant. 2016;22(4):710–6. 10.1016/j.bbmt.2015.10.009. 26475726 10.1016/j.bbmt.2015.10.009 PMC4801753

[28] Penack O, Marchetti M, Ruutu T, Aljurf M, Bacigalupo A, Bonifazi F, et al. Prophylaxis and management of graft versus host disease after stem-cell transplantation for haematological malignancies: updated consensus recommendations of the European Society for Blood and Marrow Transplantation. Lancet Haematol. 2020;7(2):e157–67. 10.1016/s2352-3026(19)30256-x. 32004485 10.1016/S2352-3026(19)30256-X

